# Constructing a Visible-Active CoFe_2_O_4_@Bi_2_O_3_/NiO Nanoheterojunction as Magnetically Recoverable Photocatalyst with Boosted Ofloxacin Degradation Efficiency

**DOI:** 10.3390/molecules27238234

**Published:** 2022-11-25

**Authors:** Pooja Dhiman, Gaurav Sharma, Abdullah N. Alodhayb, Amit Kumar, Garima Rana, Thandiwe Sithole, Zeid A. ALOthman

**Affiliations:** 1 International Research Centre of Nanotechnology for Himalayan Sustainability (IRCNHS), Shoolini University, Solan 173229, India; 2 Department of Chemistry, College of Science, King Saud University, Bldg. #5, Riyadh 11451, Saudi Arabia; 3 Department of Chemical Engineering, University of Johannesburg, P.O. Box 17011, Doornfontein 2088, South Africa; 4 Department of Physics and Astronomy, College of Science, King Saud University, Riyadh 11451, Saudi Arabia

**Keywords:** photocatalysis, Z-scheme, magnetic, water treatment, cobalt ferrite, bismuth oxide

## Abstract

Constructing visible-light-active Z-scheme heterojunctions has proven fruitful in enhancing the photocatalytic activity of photocatalysts for superior water clean-up. Herein, we report the fabrication of a CoFe_2_O_4_@Bi_2_O_3_/NiO (CBN) Z-scheme nanoheterojunction. The obtained CBN heterojunction was used for visible-light-assisted degradation of ofloxacin (OFL) in water. The OFL degradation efficiency achieved by the CBN heterojunction was 95.2% in 90 min with a rate constant of *k_app_* = 0.03316 min^−1^, which was about eight times that of NiO and thirty times that of CoFe_2_O_4_. The photocatalytic activity of a Bi_2_O_3_/NiO Z-scheme heterojunction was greatly enhanced by the visible activity and redox mediator effect of the cobalt ferrite co-catalyst. Higher charge-carrier separation, more visible-light capture, and the Z-scheme mechanism in the Z-scheme system were the important reasons for the high performance of CBN. The scavenging experiments suggested ^●^O_2_^−^ as an active species for superior OFL degradation. The possible OFL degradation pathway was predicted based on LC-MS findings of degradation intermediate products. The magnetic nature of the CBN helped in the recovery of the catalyst after reuse for six cycles. This work provides new insights into designing oxide-based heterojunctions with high visible-light activity, magnetic character, and high redox capabilities for potential practical applications in environmental treatment.

## 1. Introduction

In the past few decades, rapidly growing industrialization has led to severe environmental pollution issues. One of them is the scarcity of potable water. A number of organic and inorganic pollutants discharged through the industrial sector or day-to-day human activities are continuously entering our aquatic systems, making this issue worse [1,2]. A major contribution comes from usage of drugs or pharmaceuticals due to their high water solubility issues, which creates severe health issues [3]. Most common pharmaceuticals include chemotherapeutic agent antibiotics, which are used to restrain or kill numerous microbes for human and animal well-being [4,5]. The fluoroquinolone family is among the most widely used antibiotics, and their frequent usage has led to water contamination and associated serious health hazards. Ofloxacin (OFL), an antibiotic in this class, is generally used to treat various types of bacterial infections, but is also a source of water pollution [6,7]. OFL has been identified as a serious pollutant and detected in hospital water (35 µg L^−1^), sewage water (31.7 µg L^−1^), surface water (0.5 µg L^−1^), and municipal treatment plants (1.8 µg L^−1^) [8]. The residual OFL in the environment, especially in water sources, can reach animals and humans and cause development of drug resistance for bacteria, increasing chances of antibiotic-resistance genes [9].

Thus, it is fundamental to develop effective technologies to remove contaminants from water resources. As antibiotics are generally resistant to conventional treatment methods and biological oxidation, more efficient methods are required for pharmaceutical waste water [10,11,12]. Semiconductor photocatalysts, particularly hetero-structured photocatalysts, have emerged as promising candidates for high-performance environmental remediation [13,14]. A number of wide-bandgap semiconductors have been thoroughly explored for photocatalytic performance against different organic pollutants. Some of the potential candidates are ZnO [15], TiO_2_ [16], SnO_2_ [17], etc. However, their bare usage is slightly limited, as most of them possess catalytic efficiency stimulated only by UV light. Single photocatalysts suffer from poor stability, low surface area, high recombination, poor charge-transfer capacity, and low quantum efficiency [18]. In this direction, Z-scheme heterojunction construction can significantly improve the separation of charge carriers and also maintain the strong redox capability of the semiconductors for enhanced photocatalytic efficiency [19,20]. 

Bismuth-based materials have been employed in a number of studies recently because they can have their physical and chemical properties expertly tailored by manipulating their microstructure [21]. Bi_2_O_3_, a narrow-bandgap semiconductor with a bandgap ranging from 2–2.8 eV, is a new photocatalyst involved in the photodegradation of numerous drugs and dyes [22,23]. Bi_2_O_3_ shows different properties and applications owing to the different phase compositions of monoclinic, tetragonal, hexagonal, and cubic phases [24]. The practical applicability of Bi_2_O_3_ as a catalyst is slightly limited because of the frequent and fast recombination rate of charge carriers. To improve the visible-light activity of Bi_2_O_3_ and to reduce the charge recombination of charge carriers, it is suggested to make a composite with other organic or inorganic compounds [25]. Till date, Bi_2_O_3_ has reportedly been combined with a number of materials to enhance its the photocatalytic performance. Recently, Bi_2_O_3_/Bi_2_MoO_6_, a binary photocatalyst, has been reported for the degradation of phenol with 96.4% degradation efficiency in a 180 min experiment [26]. Recently, Bi_2_O_3_ has been efficiently combined with Zn_3_In_2_S_6_ [27], TiO_2_ [22], Co_3_O_4_/Bi_4_O_7_ [28], ZnO [29], CeO_2_ [30], MoS_2_ [31], etc. for the degradation of various water pollutants. 

NiO is a well-known semiconductor with a rock salt structure and commonly finds use in catalysts, gas sensors, solar devices, batteries, composites, and ceramic materials [32]. Nickel oxide shows excellent adsorption and catalytic properties, and possesses excellent robustness and light sensitivity [33]. The formation of a heterojunction between Bi_2_O_3_ and NiO can limit their flaws while improving their photocatalytic properties. Being less reusable and requiring a separating process, which is not cost-effective and may result in other secondary pollution, is the other difficult problem with the conventional and heterojunction photocatalysts. Making these heterojunctions magnetic can result in an efficient solution without degrading their photocatalytic efficacy [34]. Using spinel ferrites as co-catalysts or redox mediators has been a popular idea for effective charge-carrier transfer in the Z-scheme. The most popular spinel ferrites that are being explored in photocatalysis are Mn, Cu, Ni, and Co ferrites, having some advantages as compared to traditional Fe_3_O_4_ and Fe_2_O_3_ systems [35]. Among spinels, cobalt ferrite is one of the most promising magnetic oxides, with better chemical stability and separation capability due to modest saturation magnetization and a high coercivity value [36,37]. Using spinel ferrites as co-catalysts or redox mediator has been a popular idea for effective charge-carrier transfer in the Z-scheme. In addition, ferrites have attractive properties that make them suitable for biomedicine, catalysis, and environmental clean-up [38]. Moreover, the magnetic nature of the ferrites makes them easily recoverable after the degradation process. 

Motivated by the promising properties of Bi_2_O_3_, NiO, and spinel ferrites, we herein prepare a CoFe_2_O_4_@Bi_2_O_3_/NiO Z-scheme heterojunction for visible-light-driven photodegradation of ofloxacin antibiotics in the water system. In this work, we have systematically explored the structural, optical, and magnetic properties of the heterojunction. The photodegradation experiments were performed under various operational parameters with some variation, and a suitable pollutant degradation mechanism was proposed. In addition, the heterojunction was also tested under natural solar light and in river water. 

## 2. Results

### 2.1. Structural Analysis of Prepared Catalysts

**Figure 1:**
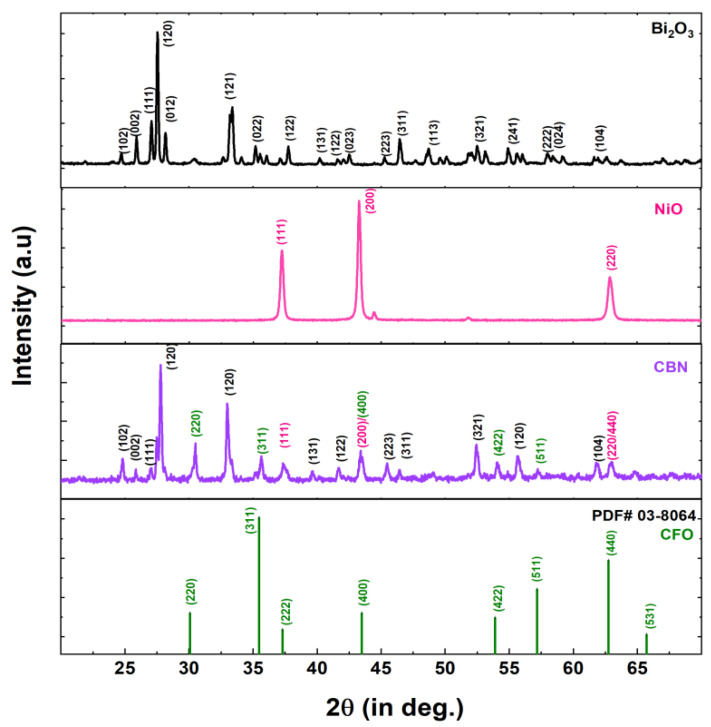
X-ray diffraction pattern for Bi_2_O_3_, NiO, and CBN samples.

CBN and bare oxides are characterized for structural analysis. The XRD patterns for all synthesized catalysts are shown in Figure 1. The XRD pattern of Bi_2_O_3_ shows the formation of a pure phase structure with (102), (002), (111), (120), (012), (121), (022), (112), (131), (122), (023), (223), (311), (113), (321), (241), (222), (024), and (104) indexed peaks, respectively, in accordance with standard JCPDS file #41-1449. This corresponds to the monoclinic crystallization of Bi_2_O_3_ [39]. 

Similarly, the NiO XRD pattern is indexed for (111), (200), and (220) planes, respectively, corresponding to the face-centered cubic structure in accordance with JCPDS file #04-0835 [40]. The observed hkl peaks (102), (002), (111), (120), (121), (223), (321), (241), and (104) in the case of CBN are assigned to the Bi_2_O_3_ structure. The presence of (111), (200), and (220) peaks corresponds to the NiO phase. The observed peaks (220), (311), (400), (422), (511), and (440) are assigned to the cubic phase structure of cobalt ferrite [41]. The presence of a characteristic peak for Bi_2_O_3_, NiO, and CFO confirms the successful formation of composites and the retention of the pure structure of bare samples.

### 2.2. Microstructural and FTIR Analysis

**Figure 2:**
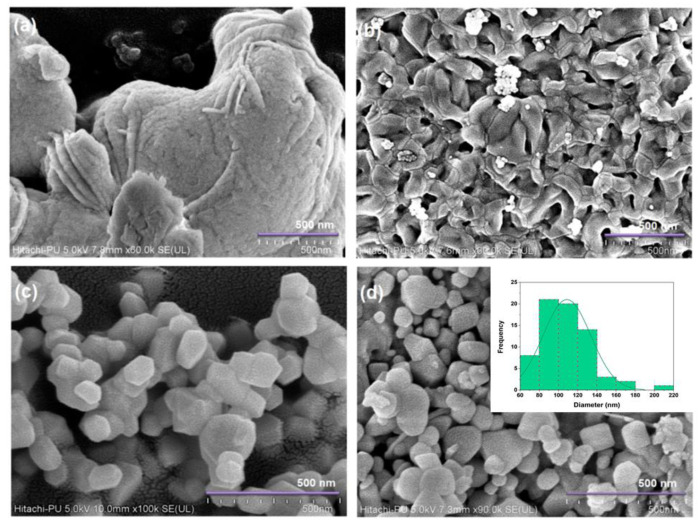
SEM images for (**a**) Bi_2_O_3_, (**b**) NiO, (**c**) CFO, (**d**) CBN; inset shows the grain size distribution for CBN photocatalyst.

The surface morphology of the synthesized catalysts is studied by analyzing SEM images. For comparison purposes, all SEM images are recorded at the same scale of magnification. Figure 2a–d presents the SEM image for the Bi_2_O_3_, NiO, cobalt ferrite (CFO), and CBN heterojunction. The bare Bi_2_O_3_ exhibits a dense and quite stacked morphology (sheet-like), while NiO nanoparticles are accompanied by a uniform and interconnected morphology of grains. The cobalt ferrite (CFO) nanoparticles have uniform distribution of grains with clear visibility. In the case of CBN, small, medium, and some large grains can be visualized. This is further verified by analyzing the grain size distribution plot (inset Figure 2d) which demonstrates the wide distribution of the grains in the CBN sample. The average grain size appears to be 100–120 nm. This is expected because of the different grains of constituent oxides of CBN. The junction shows intimate contact between the semiconductors, which is beneficial for rapid charge-transfer. 

**Figure 3:**
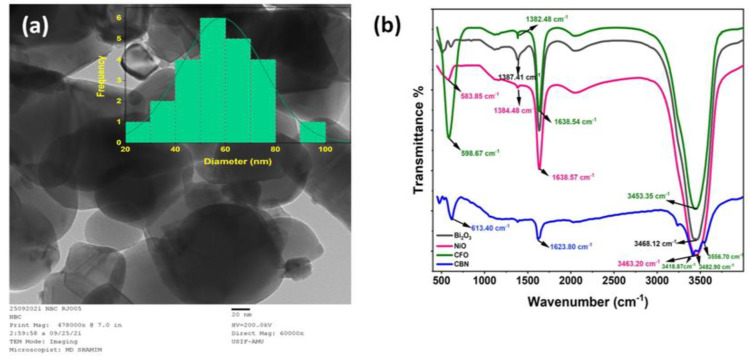
TEM images for (**a**) CBN; inset shows the histogram for particle size distribution of CBN sample (**b**) FTIR spectra for Bi_2_O_3_, NiO, CFO, and CBN samples.

The microstructural information of the CBN photocatalyst was analyzed using transmission electron microscopy. The TEM image of CBN is shown in Figure 3a. Micrographs reveal that NBC is composed of nanomaterials. The different shape and size of the nanomaterials are confirmed by micrographs, which are assigned to the different dimensions of the constituent oxides (CFO, NiO, and Bi_2_O_3_). The particle size-related information is collected by plotting a histogram for particle size distribution (inset Figure 2a). The particle size histogram reveals that the particles exist in the range of 60–200 nm, and the average particle size is found to be 110 nm.

The information on the functional groups and the bonding present in all catalysts is gathered using Fourier transform infrared spectroscopy (FTIR). Figure 3b demonstrates the FTIR spectra for all catalysts recorded in the 400–4000 cm^−1^ wavenumber range using the KBr pellet method. The peaks at 3468.12 cm^−1^ in the spectra for Bi_2_O_3_ (black color) indicate the stretching vibrations of the O-H bond [42]. The peaks observed at 837.20 and 1125 cm^−1^ are assigned to the Bi-O (M-O) vibrations [43]. The green color spectrum presents the FTIR pattern for the CFO sample. The observed band near 598.67 cm^−1^ is due to the M-O bond vibrations in the tetrahedral symmetry of the complexes, which is supposed to be one of the characteristic peaks of spinel ferrites [44]. The characteristic peak for Ni-O bond vibrations occurs at 583.85 cm^−1^ (pink color), which matches well with the literature [45]. The other IR bands observed around 1638.57 cm^−1^ and 3463.20 cm^−1^ are in all samples, and are due to symmetric and asymmetric vibrations of the C=O group and stretching vibrations of the O-H functional group. The peaks at 475.03 cm^−1^ and 613.40 cm^−1^ in the CBN IR spectrum (blue color) show different M-O bond vibrations. The peaks observed at 1623.80 are due to vibrations of the C=O group. However, the observed broad band consists of 3243.16, 3424.76, 3481.00, and 3549.00 cm^−1^ peaks which arise due to the moisture developed on the surface of the sample due to KBr method. The results confirm the presence of different M-O vibrations in the composite, which confirms the formation of the heterojunction.

### 2.3. XPS Analysis

**Figure 4:**
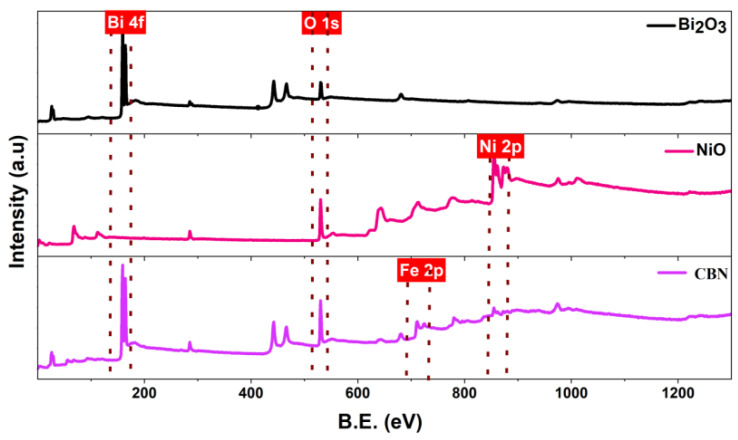
X-ray survey scan spectrum for all synthesized samples.

The X-ray photoelectron spectroscopy (XPS) technique has been used to investigate the chemical states, element confirmation, element environment, and presence of any defects on the surface of the catalyst. Nevertheless, XPS is a crucial tool for exploring charge transfer in heterojunctions. Figure 4 shows the X-ray survey scan spectrum for Bi_2_O_3_, NiO, and CBN photocatalysts. Bi_2_O_3_ XPS spectra confirm the presence of Bi 4f, Bi 4d, and O 1s states in the sample. Similarly, Ni 2p and O 1s states are confirmed for the NiO sample. On comparing the CBN spectra with the bare samples, a slight shift can be observed in the Ni 2p, Bi 4f, and O1s states. The shifting is an indication of the successful formation of the heterojunction. The elemental percentage derived from the XPS spectra of CBN photocatalyst is listed in Table S1. More information can be gathered by analyzing the core-level spectra of individual element states.

**Figure 5:**
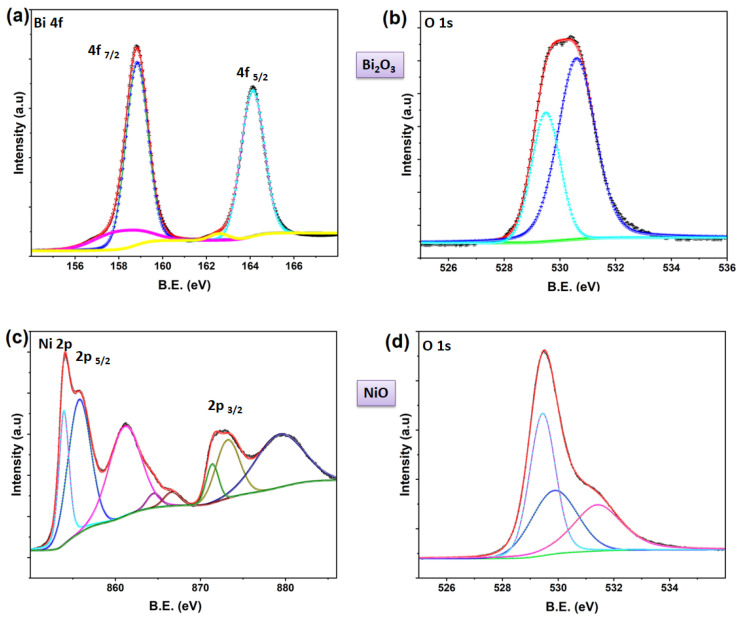
De-convoluted core level spectra for (**a**,**b**) Bi_2_O_3_ (Bi 4f and O 1s spectra), (**c**,**d**) NiO (Ni 2p and O1s spectra).

**Figure 6:**
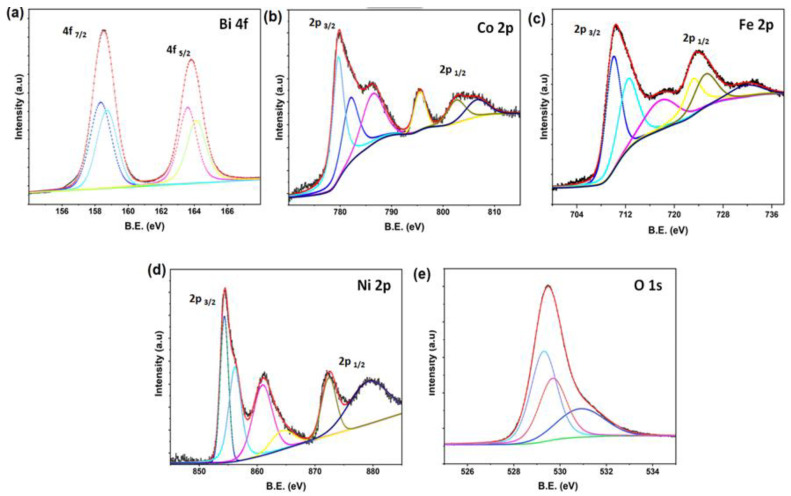
De-convoluted core level spectra for CBN (**a**) Bi 4f, (**b**) Co 2p, (**c**) Fe 2p, (**d**) Ni 2p, and (**e**) O 1s spectra).

The XPS core-level spectra for Bi_2_O_3_ photocatalyst are shown in Figure 5a,b. Bi4f de-convoluted spectra are shown in Figure 5a. The spectrum is fitted for Bi 4f_7/2_ and Bi 4f_5/2_ peaks. The two major peaks are observed at 158.82 eV and 164.15 eV, respectively. The 5.33 eV peak separations between the two observed peaks match with the literature [46] and confirm the presence of the +3 valence state of Bi in the synthesized Bi_2_O_3_ nanoparticles. Figure 5c,d depicts the XPS core-level spectrum for NiO catalysts. A Ni 2p spectrum is fitted with 853.39, 855.85, 861.25, 864.55, 866.75, 871.34, 873.19, and 879.59 eV, respectively. The peaks at 853.39 eV and 873.19 eV correspond to the Ni 2p_3/2_ and 2p_1/2_ peaks, respectively. However, 861.25 and 879.59 eV binding energy peaks closely match the position of satellite peaks [47]. The presence of metallic Ni can be denied due to absence of any peaks below the binding energy of 852 eV. Other peaks could be caused by Ni_2_O_3_ and Ni(OH)_2_ [48]. However, the possibility of other Ni oxide phases is not supported by the XRD results. Similarly, O1s is also de-convoluted with three peaks, in which 529.46 eV is assigned to the Ni-O bonding. The other two peaks, residing at 530.1 eV and 531.41 eV, are devoted to the oxygen vacancies and molecular oxygen traces adsorbed on the surface of the catalyst [49]. More information can be gathered from the XPS spectra of the CBN sample as shown in Figure 6a–e. Bi 4f spectra are fitted with two sets of peaks for 4f_7/2_ and 4f_5/2_. However, there is a slight shift in both peaks towards the left when compared to the Bi 4f spectra for the Bi_2_O_3_ sample. Lowering of the binding energy is an indication of successful junction formation, as the electron transfer may lead to surface densities. Co2p spectra shows peaks at binding energies of 779.62, 782.12, 786.68, 795.61, 802.56, and 806.75 eV, respectively. The peaks at 779.62 and 795.61 eV are assigned to the Co^2+^ state of cobalt in the CBN sample [50]. The fitted peak under major peaks denotes the tetrahedral and octahedral environment of Co in the sample. The other peaks observed are identified as satellite peaks. The Fe 2p spectra also confirm the presence of Fe in 3+ states. On comparing the O1s spectra of the CBN sample with the O1s spectra of NiO and Bi_2_O_3_ samples, a major shift in the peaks are observed. The spectra for CBN are fitted with three peaks. The lowest binding energy peak resides at 529.29 eV, slightly shifted to the left-hand side with respect to the NiO and Bi_2_O_3_ catalysts. Overall, the XPS data show that charge transfer takes place through the junction in the CBN sample.

### 2.4. Raman Spectroscopy and Magnetic Studies

**Figure 7:**
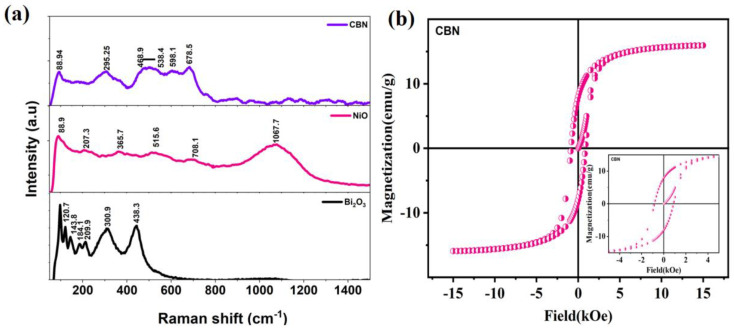
(**a**) Vibrational modes observed for Bi_2_O_3_, NiO, and CBN catalysts in Raman spectra (**b**) Hysteresis loops for CBN, inset: Zoomed in around origin of M-H curve.

Figure 7a shows the Raman spectra for Bi_2_O_3_, NiO, and CBN catalysts. According to group theory, Bi_2_O_3_ has C_2h_ symmetry and 30 theoretically active Raman modes (15Ag + 15Bg). However, experimentally, only 15 or fewer Raman active modes are observable as per the literature [51,52]. In our case, peaks are observed at 120.7, 143.8, 184.1, 209.9, 300.9, and 438.3 cm^−1^, respectively, which matches well with those reported for α-Bi_2_O_3_ Raman modes [53]. The results are in good agreement with the XRD results, as well. The mode at 120.7 cm^−1^ is due to the Ag symmetry caused by bismuth atom participation, whereas the higher-wavenumber band at 143.8 cm^−1^ is caused by the movement of bismuth and oxygen atoms. All other higher-wavenumber modes are due to the displacement of oxygen atoms in Bi_2_O_3_. The lack of peaks above 500 cm^−1^ indicates that no complex structure is formed in Bi_2_O_3_. The NiO Raman spectrum contains bands at 88.9, 207.3, 365.7, 515.6, 708.1, and 1067.1 cm^−1^. The band residing at 708.1 cm^−1^ refers to the second-order TO mode, and the band at 1067.1 is due to LO modes. The band at 515.6 cm^−1^ is assigned to the first-order LO mode [54]. The other first-order TO mode is not clearly visible due to the flattening and broadening of the Raman bands. This type of broadening of LO and TO modes has been also reported in the literature [55]. The Raman spectrum for CBN reflects the presence of various Raman modes at 88.94, 295.25, 468.9, 538.4, 598.1, and 675.5 cm^−1^ respectively. Some of the modes overlap with the Raman modes predicted for NiO, Bi_2_O_3_, and CFO. The presence of a high-frequency mode at 675.5 cm^−1^ may be assigned to the vibration of oxygen atoms in M-O_4_-type environments, indicating the confirmation of CFO in the composite material. Overall, Raman analysis confirms the presence of all Raman modes and defects belonging to NiO, Bi_2_O_3_, and CFO in CBN. Further, the synthesized catalyst has been explored for its magnetic properties, as magnetic separation helps in the recovery of the catalyst after reuse. To know the magnetic behavior of the catalyst, hysteresis loops for the heterojunction are recorded by performing the experiment on VSM. Figure 7b presents the room-temperature hysteresis curves of the heterojunction catalyst. The M-H loops have been recorded under the application of an external magnetic field strength of 15,000 Oe. The M-H curve shows clear magnetic behavior of the CBN catalyst. The catalyst possesses a saturation magnetization of 16.20 emu/g along with a coercive field of 977 Oe (inset Figure 7b). The observed magnetic parameters suggest that the catalyst is quickly recoverable and can be restored after the degradation process. 

### 2.5. Complete Band Structure Determination 

**Figure 8:**
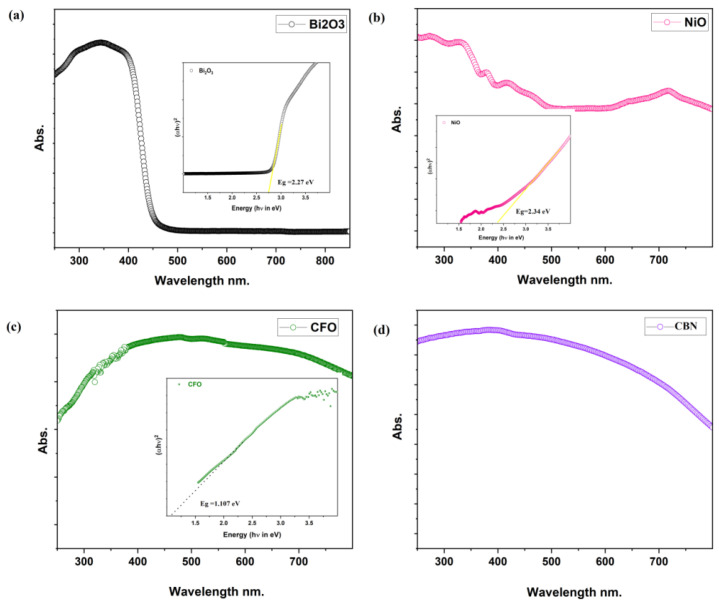
(**a**–**d**). Absorbance spectra for Bi_2_O_3_, NiO, CFO, and CBN catalysts. **Inset:** Tauc plot for bandgap calculation.

**Figure 9:**
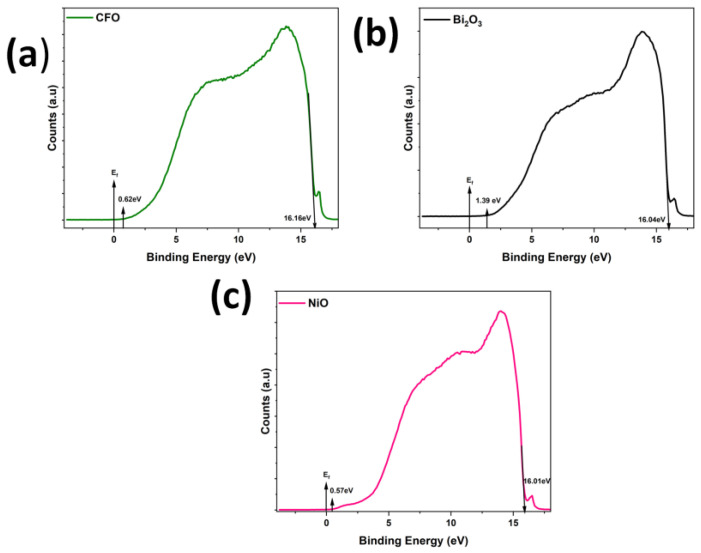
(**a**–**c**) UPS spectra for CFO, Bi_2_O_3_, and NiO.

The optical behavior of bare and composite catalysts is studied using UV–visible spectroscopy. The bandgap of the bare catalysts is determined from Tauc plotting using absorbance spectrum data. Figure 8a,d shows the absorbance spectra for all synthesized catalysts. The absorbance spectrum for Bi_2_O_3_ indicates low absorbance in the visible region in comparison to NiO and CFO catalysts. However, the heterojunction CBN has a much higher absorbance in the visible region than bare NiO and Bi_2_O_3_, allowing it to make full use of visible light. The optical bandgap energies for Bi_2_O_3_, NiO, and CFO are estimated using (αhν)^2^ vs. hν plots, and are found to be 2.75, 2.34, and 1.10 eV, respectively. The bandgap obtained for Bi_2_O_3_ is a close match with the reported values in the literature [56,57]. The bandgap for CFO is also in close agreement with the findings observed by Sumathi et al. [58]. The bandgap of CBN (Figure S1) was found to be 1.57 eV which ensures high visible-light absorption. The position of conduction, as well as valence bands, in the heterojunction play a significant role in determining the charge transfer of carriers between other semiconductor oxides. UPS (ultraviolet photoelectron spectroscopy) is an important tool to have better insight into the electronic properties of materials. UPS is utilized for the determination of valence band positions in all bare semiconductor catalysts. The photoemission spectra for Bi_2_O_3_, NiO, and CFO are recorded using ultra-photoelectron spectroscopy with He I as the light source (21.22 eV). Figure 9a–c presents the UPS spectra for all bare photocatalysts. After the calibration, Fermi level of 0 V versus RHE equals −4.44 eV versus the vacuum level. The valence band maxima, conduction band minima, and Fermi energies are evaluated according to the standard method reported by Mallick et al. [59]. The calculated valence band maxima for CFO, Bi_2_O_3_, and NiO are 1.24 eV, 2.13 eV, and 1.34 eV, respectively. The obtained values are 0.13 eV (CFO), −0.6 eV (Bi_2_O_3_), and −1.0 eV (NiO), respectively. 

### 2.6. Degradation Performance against Ofloxacin

**Figure 10:**
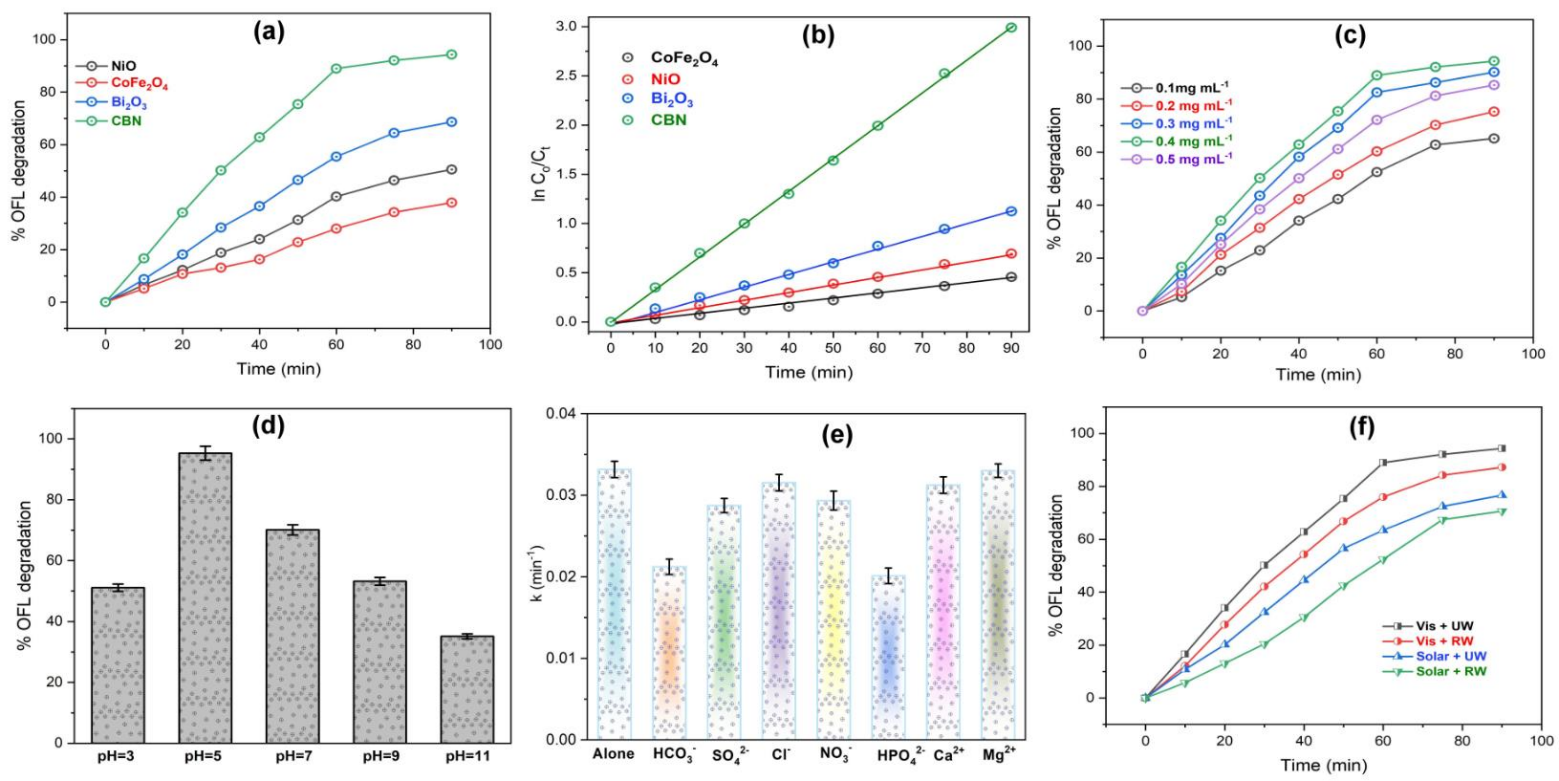
(**a**) Photocatalytic removal of OFL (**a**) Photodegradation under visible light (**b**) Corresponding pseudo-first-order kinetic plots; effect of reaction parameters on OFL removal under visible light with CBN photocatalyst (**c**) Catalyst dosage (**d**) Initial solution pH (**e**) Common electrolytes (**f**) Solar light and water source [photocatalyst] = 0.4 mg/mL; [pollutant] = 10 mg L^−1^, initial pH = 5, temperature = 30 ± 0.5 °C.

The prepared bare catalysts and heterojunction were tested for their efficiency in the visible-light-assisted degradation of ofloxacin. Ofloxacin is used to cure bacterial infections in humans. The chemical structure of ofloxacin is shown in the inset of Figure 10a. The plain photolysis of OFL under visible light (Figure S2) shows a mere 3.15% removal in 90 min, suggesting stability in aqueous medium and resistance to degradation. The adsorption potential of samples was tested in a 60 min experiment in the dark (Figure S3). Cobalt ferrite shows 17.3% removal of OFL via adsorption in 60 min, followed by NiO (11.5%) and Bi_2_O_3_ (10.2%). The junction CBN also shows a poor adsorption potential of 13.3%. This was further tested by a 60 min dark experiment followed by light exposure for 90 min (Figure S4). The OFL removal order is CBN (85.2%) > Bi_2_O_3_ (58.5%) > NiO (40.7%) > CoFe_2_O_4_ (40.7%) with a total experiment time of 150 min. Figure 10a shows the OFC degradation plots for a 90 min experiment with direct exposure to visible light. The order of performance for OFL removal is CBN (95.2%) > Bi_2_O_3_ (68.6%) > NiO (50.5%) > CoFe_2_O_4_ (37.8%). Because of the lower bandgap, NiO performs poorly due to increased electron–hole recombination. Because of the high visible absorption and improved charge separation via Z-scheme transfer, the performance improves manifolds on junction formation [60]. The corresponding pseudo-first-order kinetic plots corresponding to the degradation curves are shown in Figure 9b. The calculated rate constants are CBN (0.03316 min^−1^) > Bi_2_O_3_ (0.01152 min^−1^) > CFO (0.00769 min^−1^) > NiO (0.00113 min^−1^). The OFL degradation rate was nearly eight times that of NiO, and thirty times that of CoFe_2_O_4_. As was observed, due to the average adsorption potential of the photocatalysts, the role of adsorption is not very pronounced in overall photocatalytic OFL removal. This was supported by the 1 h adsorption + 90 min light exposure experiment. The performance of all the catalysts is lower in this condition than in direct exposure experiments, but not to a substantial extent. The adsorbed pollutant (during the 1 h dark experiment) further blocks the light exposure to some extent, which decreases the photocatalytic activity. When the experiment is conducted under direct exposure, the pollutant is adsorbed and is quickly degraded by the surface-generated radicals. As observed from the UV–visible spectra, the CBN heterojunction shows very high visible-light activity which improves the photocatalytic performance. The cobalt ferrite nanoparticles not only provide magnetic character to the photocatalyst but also act as co-catalysts or redox mediators for facile Z-scheme transfer. The heterojunction formation between Bi_2_O_3_ and NiO provides optimum visible-light absorption, high charge-transfer capacity and highly diminished recombination of photogenerated electron–hole pairs. These results have been supported by PL measurements discussed in later section. Because errors in photocatalytic reactions are possible, experiments were carried out in triplicate with error calculation (error bars). The calculated error in this study, however, was less than 4%. Hence, the standard deviation was not plotted here to maintain the simplicity of the information provided by the graphs. The photocatalytic mechanism is discussed in detail in subsequent sections.

#### Operational Parameters

The effect of various operational parameters and conditions on pollutant degradation is important to study for practical and best performance optimization. Figure 10c shows the effect of CBN dosage on OFL removal under visible light. The degradation efficiency for 0.1 mg mL^−1^ is 65.1%, which increases with increasing dosage up to 0.3 mg mL^−1^ (95.2%), falls marginally at 0.4 mg L^−1^, and falls up to 85.2% with further high catalyst content. The decrease in the photocatalytic performance for the higher catalyst loading is attributed to the greater turbidity of the solution and increase in scattering sites with higher catalyst dose. On the other hand, due to the low catalyst dosage, photocatalytic performance is also low, which is expected due to limitations of active sites in the degradation process. The antibiotic chemistry of an aqueous medium depends largely on the initial solution pH. Figure 10d shows the effect of initial pH on OFL degradation with CBN catalyst. The highest OFL removal was achieved at pH = 5. The pKa values for OFL are 5.97 (for –COO^−^ group) and 9.28 for piperazinyl ring [13]. At a starting or working pH of 5, the dominant form is OFL+. The pzc for the CBN heterojunction photocatalyst was discovered to be 4.6, implying that the surface would be negatively charged at pH = 5. Both the antibiotic and CBN are negatively charged at alkaline pH, which limits the interactions and also the attack of reactive oxygen species. In addition, the HCO_3_^−^ ions are formed at alkaline pH via atmospheric CO_2_ and, hence, quenching of hydroxyl radicals takes place [61].

In real-water conditions, various electrolytes exist that may interfere with the generation of reactive species and, hence, the photodegradation efficiency. To simulate the effect, various electrolytes with a concentration of 10 mM were added during OFL degradation with CBN and the results are shown in Figure 10e. The order of electrolytes in order of detrimental effect on OFL degradation rate is as follows: HPO_4_^2−^ (0.02011 min^−1^) > HCO_3_^−^ (0.02123 min^−1^) > SO_4_^2−^ (0.02873 min^−1^) > NO_3_^−^ (0.02954 min^−1^) > Cl^−^ (0.03155 min^−1^). Cl^−^ ions exhibit a marginal fall in OFL degradation as they scavenge ^●^OH radicals to some extent. The OFL removal rate was lowest in the presence of HPO_4_^2−^ because it tends to compete with radicals for adsorption onto surfaces. In addition, the HPO_4_^2−^ ions would increase the repulsive forces for pollutant molecules when present on the catalyst surface. The HCO_3_^−^ ions also decrease the OFL degradation rate as they tend to scavenge ^●^OH radicals to generate less-active CO_3_^−●^. The CO_3_^−●^ radicals are more selective, but with less reactivity they decrease the degradation rate. Mg^2+^ ions have a negligible effect on OFL removal rate (0.03302 min^−1^). On the other hand, the rate falls with Ca^2+^ ions (0.03124 min^−1^) as they can form co-ordination complexes with multi-functionality organic molecules such as drugs. The effect of various electrolytes on the OFL degradation with CBN infers that catalyst can work under various conditions with good efficacy which is necessary for practical field applications. 

The OFL removal was also tested under conditions of solar light and river water (RW), and results are shown in Figure 10f. 

The CBN photocatalyst shows OFL removal as follows: CBN + UW + visible (95.2%) > CBN + RW + visible (87.3) > CBN + UW + solar (76.6%) > CBN + RW + solar (70.5%). In river water, the OFL removal efficiency falls because it contains several ions and dissolved organic matter that hinder light absorption and generation of active oxygen species. The lower rate in tap water is because of the presence of interfering ions and organic matter. Even under solar light, the CBN heterojunction performs well, but not as well as visible light as the majority of solar light is infrared. However, because the photocatalyst has a high removal efficiency in natural sunlight and river water, it is suitable for practical environmental clean-up applications.

### 2.7. Pollutant Degradation Pathway and Photocatalytic Mechanism

**Figure 11:**
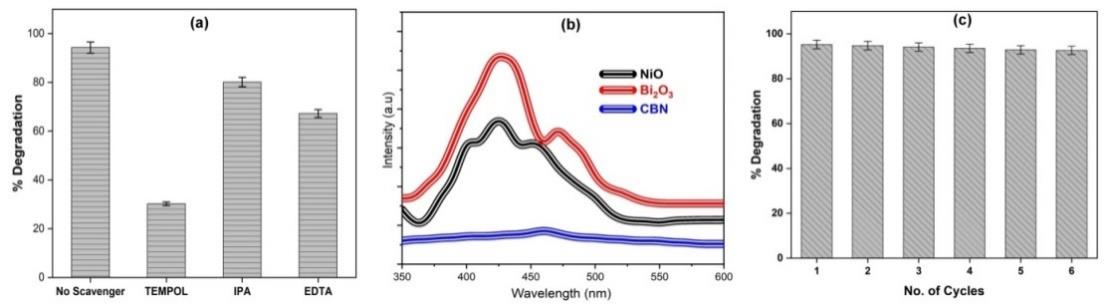
(**a**) Effect of scavengers on photocatalytic removal of OFL with CBN heterojunction (**b**) Photoluminescence spectra for synthesized photocatalysts (**c**) Reusability studies for OFL removal under visible light with CBN photocatalyst [photocatalyst] = 0.3 mg/mL; [pollutant] = 10 mg L^−1^, initial pH = 5, temperature = 30 ± 0.5 °C.

To understand the mechanism of degradation of ofloxacin with a CBN photocatalyst, scavenging experiments were performed for the identification of reactive species in the photodegradation process. The scavengers used in the experiments were 4-hydroxy-2,2,6,6-tetramethylpiperidin-1-oxyl (TEMPOL), ethylene diamine tetraacetic acid (EDTA), and iso-propyl alcohol (IPA), scavenging ^●^O_2_^−^, h^+^, and ^●^OH radicals, respectively. Figure 11a shows that the OFL degradation falls substantially to 30.3% upon addition of TEMPOL, suggesting that ^●^O_2_^−^ radicals are the main active species. With EDTA, the degradation reaches 67.1%, suggesting some contribution from photogenerated holes, too. As such, the scavenging experiment findings suggest that, during OFL degradation with CBN, ^●^O_2_^−^ and h^+^ are the major reactive species. The detailed photocatalytic mechanism of the band alignment and possible charge-transfer routes is discussed in subsequent sections.

**Scheme 1:**
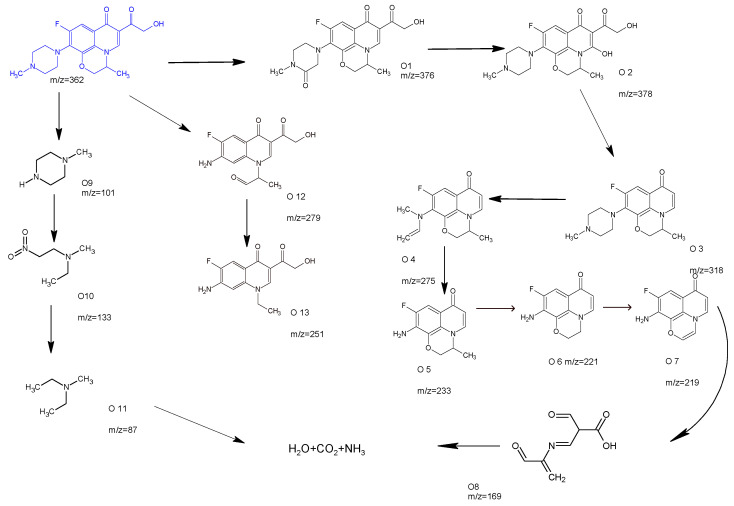
Possible OFL degradation pathway.

Further, LC-MS was employed to detect and analyze the OFL degradation intermediates, and a possible degradation route was proposed as shown in Scheme 1. The first typical pathway involves attack by holes/^●^O_2_^−^ on the piperazine ring in a parent molecule (m/z = 362) to form intermediates O1 (m/z = 376) and O2 (m/z = 378). Further oxidation leads to cleavage of the C-C bond in the ring, N-dealkylation, and formation of O3 (m/z = 318). The O3 intermediate forms O4 (m/z = 275) via opening of the piperazine ring. The further dealkylation of O4 leads to O5 (m/z = 233) and O7 (m/z = 219). The ring opening then leads to the formation of (m/z = 169). The degradation intermediates detected are in accordance with previously reported works [62,63]. In the second degradation route, the C-N bond cleavage occurs due to radicals to form intermediates O9 (m/z = 101), and further cleavage of the piperazine ring forms O10 (m/z −133) and O11 (m/z = 87) [4]. In the third route, the parent molecule (m/z = 362) leads to formation of O12 (m/z = 279) with amino group removal via piperazine epoxidation. Subsequent radical attack forms product O13 (m/z = 251). The results are in agreement with previous findings [64]. The repeated attacks by the reactive oxygen species leads to formation of lower molecular weight aliphatic molecules, and mineralization in the longer run. This was further ascertained by high total organic carbon (TOC) removal (Figure S5). The order of TOC removal under visible light irradiation for a 2 h experiment using CBN as the photocatalyst was: CBN + UW + Visible (83.4%) > CBN + RW + Visible (77.6%) > CBN + UW + Solar (72.1%) > CBN + RW + Solar (62.2%). High TOC removal in the visible light, solar light, and river water matrix suggests that the CBN photocatalyst has competent mineralization capacity, which increases with longer runs. 

**Figure 12:**
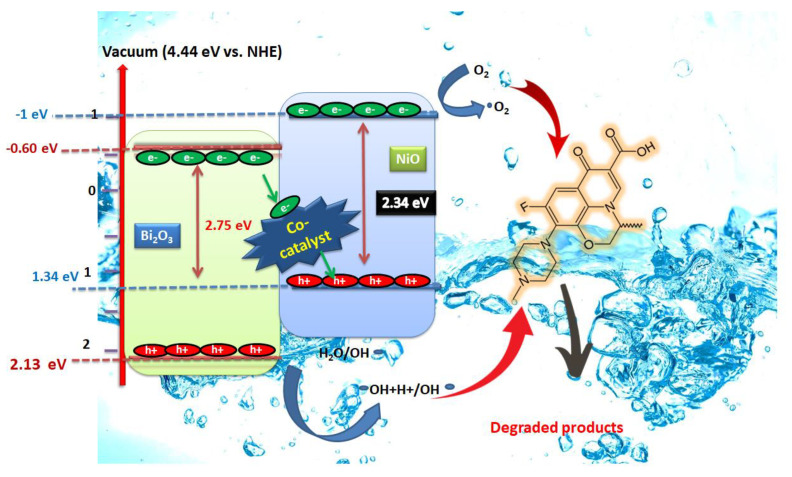
Proposed photocatalytic degradation mechanism.

The superior photocatalytic performance needs to be supported by the photocatalytic mechanism/charge transfer involved and the thermodynamic feasibility of the generation of radicals at the conduction and valence bands of the junction. Figure 12 shows the structural arrangement of the CBN heterojunction, depicting the possible charge-transfer routes. The CB and VB arrangements show that Bi_2_O_3_ and NiO have a staggered configuration in the heterojunction. On irradiation, both semiconductors get excited by the generation of electron–hole pairs. The structural arrangement of the bands can suggest a type-II transfer mechanism or a Z-scheme. In a typical type-II route, the electrons from CB of NiO (−1.00 eV) would move to that of Bi_2_O_3_ (−0.60 eV) and lose potential. Similarly, holes would move from the VB of Bi_2_O_3_ (2.13 eV) to the less-positive VB of NiO (1.34 eV). Thus, both electrons and holes would be separated, but would lose potential to oxidize the pollutants via poor generation of radicals. Thus considering the superior performance of the heterojunction, the type-II mechanism is ruled out and the Z-scheme mechanism is proposed. In a Z-scheme route, the photogenerated electrons migrate from the CB of Bi_2_O_3_ (−0.60 eV) towards the VB of NiO (+1.34 eV) and recombine. Thus, the electrons get accumulated in high potential CB of NiO (−1.00 eV) and the holes get separated and accumulated in the VB of Bi_2_O_3_ (−0.32 eV). The separated electrons in the CB of NiO have high enough potential to generate ^●^O_2_^−^ from O_2_ because E^o^(O_2_/^●^O_2_^−^) = −0.33 eV. Thus, thermodynamically, there is a high probability of generation of superoxide radical anions. This is also supported by the scavenging experiment results. Additionally, the holes accumulated in the VB of Bi_2_O_3_ can further oxidize the pollutant directly or via generation of ^●^OH radicals via H_2_O or OH^−^ ions.

The reaction occurs at pH = 5; hence, the route via OH^−^ is ruled out. In addition, the holes on VB of Bi_2_O_3_ cannot generate ^●^OH radicals, as potential is lower than E(^●^OH/H_2_O) = 2.27 eV vs. NHE [65]. The movements of electrons are also supported by XPS measurements. The charge separation and reduced recombination in the CBN heterojunction are also supported by the photoluminescence (PL) spectra (Figure 11b). As can be seen, NiO and Bi_2_O_3_ both show very high PL intensities because of their lower bandgaps, which increase recombination. However, the PL curve is almost flattened for the CBN junction, thereby suggesting high charge separation by effective Z-scheme transfer.

The CoFe_2_O_4_ nanoparticles act as redox mediators, which support the facile indirect Z-scheme transfer of photogenerated electrons from CB of Bi_2_O_3_ towards VB of NiO as Bi_2_O_3_ → CoFe_2_O_4_ → NiO or Bi_2_O_3_ → Fe^2+^/Fe^3+^ → NiO or Bi_2_O_3_ → Co^2+^/Co^3+^ → NiO. The Fe^2+^/Fe^3+^ redox cycle can easily transfer electrons in a facile manner and thus support the separation of charge carriers. In addition, the electron transfer may take place from CoFe_2_O_4_ towards the CB of NiO to generate more ^●^O_2_^−^ radicals which boost the OFL degradation. The ferrite nanoparticles also improve the visible absorption of the junction and provide magnetic character to the photocatalyst for better separation and recovery. 

The reusability and stability of the photocatalyst is important for practical applications. Figure 11c shows the reusability of the CBN heterojunction for OFL removal with visible light for five consecutive cycles. It was observed that the OFL removal efficiency fell marginally for most of the cycles, which shows the catalyst is robust and reusable. As the catalyst has a high enough magnetization, it can be easily separated and recovered for further use via a magnetic field. 

## 3. Materials and Methods

### 3.1. Materials

Bismuth nitrate (Bi (NO_3_)_3_.5H_2_O, ≥98%; Sigma-Aldrich), nickel nitrate (Ni (NO_3_)_2_.6H_2_O, ≥97%; Sigma-Aldrich, Saint Louis, MO, USA), cobalt nitrate (Co (NO_3_)_2_.6H_2_O, 98%; Sigma-Aldrich, Delhi, India), iron nitrate (Fe (NO_3_)_3_.9H_2_O, ≥99.95; Sigma-Aldrich), and glycine (C_2_H_5_NO_2,_ ≥99.7%; Merck, Darmstadt, Germany) were used for the synthesis of catalysts. All materials used for the work under study were of analytical grade. 

### 3.2. Synthesis of Bi_2_O_3_, NiO and CoFe_2_O_4_ Nanoparticles

Bi_2_O_3_ nanoparticles were synthesized by the solution combustion method. In a simple procedure, 19.40 g of bismuth nitrate and 6.00 g of glycine were dissolved in 50 mL of double-distilled water and stirred magnetically until complete dissolution at 80 °C. The solution was allowed for stirring and heating for an hour. After that, the temperature was raised to 120 °C for complete combustion. Thereafter, fluffy material was collected and then ground into fine powder. The resulting powdered material was annealed at 500 °C for 4 h. The cooled down material can be directly used for further characterization purposes.

NiO nanoparticles were synthesized using a similar procedure to that of the synthesis of Bi_2_O_3_ nanoparticles. In this route, Ni nitrate (11.63 g) along with glycine (6.00 g) were dissolved in 50 mL of double-distilled water.

CoFe_2_O_4_ nanoparticles were also prepared using the solution combustion route by taking Co nitrate (5.82 g) and Fe nitrate (16.16 g) as oxidizers and glycine (3.00 g) as reducing agent/fuel. Details of the experiment were similar to the above methods except the calcination conditions. The annealing of the powder was done at 750 °C for 4 h. The prepared catalyst was labelled as CFO.

### 3.3. Synthesis of CoFe_2_O_4_@Bi_2_O_3_/NiO Heterojunction

In a simple procedure, the pre-synthesized 2.95 g of Bi_2_O_3_, 1.89 g of NiO, and 1.89 g of CoFe_2_O_4_ were mixed, and the mixture was grinded with the help of mortar and pestle for 5 h to obtain a homogeneous solid mixture. Further, the powdered material was annealed at 550 °C for 5 h. The powdered sample obtained was cooled to room temperature, further ground, and stored for application purposes. The synthesized photocatalyst was labeled as CBN.

### 3.4. Characterization Details of Catalysts

The synthesized catalysts were analyzed for structural information using an X-ray diffractometer (Panalytical’s X’Pert Pro, Malvern, UK) with a Cukα radiation source. The experiments were performed in the 2θ scanning range of 20–70 degrees with step size of 0.02°. The particle size of catalysts was obtained with the help of a transmission electron microscope (EM-410 LS, Philips, Amsterdam, The Netherlands). The surface morphology of the catalysts was examined using a scanning electron microscope (SEM, Hitachi, Tokyo, Japan). X-ray photoelectron spectroscopy (Thermofisher Scientific, Nexsa base, Waltham, MA, USA) was employed for the determination of elemental presence and their valence states. The metal-stretching vibrations in the synthesized catalysts were performed with the help of a Raman spectrometer (Raman Horiba, Lab RAM HR evolution). The bandgap estimation was performed by analysis of the absorbance spectra recorded using a UV-VIS-NIR spectrophotometer (Perkin Elmer, Waltham, MA, USA). The magnetic properties of the catalysts were investigated using a vibrating sample magnetometer (Micrösense, EV7) to record the room temperature M-H loop at a field strength of 15,000 Oe. Photoluminescence (PL) spectra were recorded on a FS5 spectrophotometer (Edinburgh Instruments, Edinburgh, UK). 

### 3.5. Photocatalytic Degradation Experiment

The fabricated photocatalysts were employed for the degradation of ofloxacin (OFL) at 30 ± 0.5 °C under visible-light irradiation (Xe lamp, 350 W) in a Pyrex glass reactor. A λ > 420 nm cut-off visible-light filter was used to ensure visible-light irradiation. The experiment was started with an initial catalyst dosage of 30 mg added into 100 mL containing 10 mg/L ofloxacin solution and then exposed to a light source. After regular intervals of time, 20 μL of reaction suspension was injected out using a syringe and then filtered for any unwanted catalyst particles via a 0.22 μm membrane filter. The concentration of residual OFL was analyzed using high-performance liquid chromatography (HPLC) equipment (Agilent ZORBAX Eclipse, Santa Clara, CA, USA) equipped with a C18 column and UV detector at 288 nm. Methanol (15%) and 1% formic acid in ultrapure water (85%) were used as mobile phases. The flow rate was set at 1 mL min^−1^. The OFL degradation intermediates were detected by LC-MS (Agilent 1290 UPLC) in the positive electrospray ionization (ESI) mode. The total organic carbon was analyzed using a Vario TOC analyzer (Elementar, Langenselbold, Germany). The percentage degradation of ofloxacin was calculated using the following formula [66]:

(1) $$\%\;degradation=\frac{C_{o}-C_{t}}{C_{o}}\times 100$$

where Co and Ct are the concentration of pollutant before the experiment and at time ‘t’.

The kinetics of the degradation was studied with the pseudo-first-order kinetic equation given by [67]:

(2) $$ln\frac{C_{o}}{C_{t}}=k_{app}t$$

## 4. Conclusions

In this study, CoFe_2_O_4_@Bi_2_O_3_/NiO heterojunction photocatalysts were synthesized for visible-light-assisted OFL degradation. It was found that the OFL removal rate with CBN heterojunction was about eight times that of NiO and thirty times that of CoFe_2_O_4_. The experimental results demonstrated that ^●^O_2_^−^ radicals were the main active species involved, with some contribution from photogenerated holes. It can be inferred from the optical and photoluminescence results that the CBN heterojunction showed high visible-light capture potential and highly diminished recombination of charge carriers. The XPS findings and band structure analysis suggested Z-electron transfer between Bi_2_O_3_ and NiO facilitated by CoFe_2_O_4_ as a co-catalyst/redox mediator. In terms of practical application, the OFL removal was also tested under natural sunlight at various operational parameters with the co-existing electrolytes and river water matrix, and it was discovered that the CBN heterojunction was conducive to photocatalytic activity in a variety of environments. The OFL degradation pathway was also suggested according to the intermediates detected by LC-MS analysis. The CoFe_2_O_4_ nanoparticles impart magnetic character to the catalyst, improve visible activity, and act as redox mediator for facile indirect Z-scheme transfer of photogenerated electrons from CB of Bi_2_O_3_ towards the VB of NiO as Bi_2_O_3_ → CoFe_2_O_4_ → NiO or Bi_2_O_3_ → Fe^2+^/Fe^3+^ → NiO or Bi_2_O_3_ → Co^2+^/Co^3+^ → NiO. Furthermore, the CBN heterojunction exhibited good stability and recyclability. The overall research demonstrates that efficient heterojunctions have good prospects in organic pollutant degradation, heavy metal clean-up, clean energy generation, greenhouse catalytic conversion into fuels utilizing natural sunlight, and in real-water systems.

## Data Availability

Not applicable.

## Supplementary Materials

The following supporting information can be downloaded at: https://www.mdpi.com/article/10.3390/molecules27238234/s1, Figure S1. Tauc plot for CBN heterojunction. Figure S2. Plain photolysis of OFL under visible light. Figure S3. Adsorptional removal of OFL in dark. Figure S4. Removal of OFL: 60 min dark followed by 90 min visible light exposure. Figure S5. TOC removal during OFL degradation with CBN under visible light exposure. Table S1. Elemental presence derived from XPS spectra for Bi_2_O_3_, NiO, and CBN.
