# Supplementary material for: Constructing a Visible-Active CoFe2O4@Bi2O3/NiO Nanoheterojunction as Magnetically Recoverable Photocatalyst with Boosted Ofloxacin Degradation Efficiency
Source: Molecules. 2022 Nov 25;27(23):8234. doi: 10.3390/molecules27238234 (PMC9741353; doi:10.3390/molecules27238234)
Supplement: Supplementary file 1 [file molecules-27-08234-s001.zip › molecules-1924400-supplementary.pdf]

Constructing a Visible-Active $CoFe_2O_4$@Bi_2O_3/NiO Nanoheterojunction as Magnetically Recoverable Photocatalyst with Boosted Ofloxacin Degradation Efficiency

Pooja Dhiman [1], Gaurav Sharma [1,2,3,*], Abdullah N. Alodhayb [4], Amit Kumar [1], Garima Rana [1], Thandiwe Sithole [3] and Zeid A. ALOthman [2]

[1] International Research Centre of Nanotechnology for Himalayan Sustainability (IRCNHS), Shoolini University, Solan 173229, India
[2] Department of Chemistry, College of Science, King Saud University, Bldg. #5, Riyadh 11451, Saudi Arabia
[3] Department of Chemical Engineering, University of Johannesburg, P.O. Box 17011, Doornfontein 2088, South Africa
[4] Department of Physics and Astronomy, College of Science, King Saud University, Riyadh 11451, Saudi Arabia

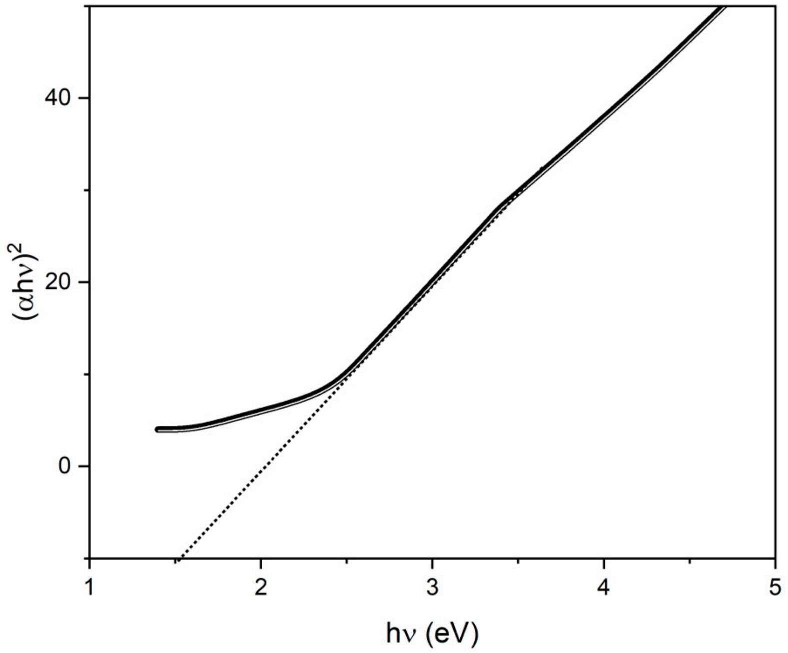

Figure S1. Tauc plot for CBN heterojunction.

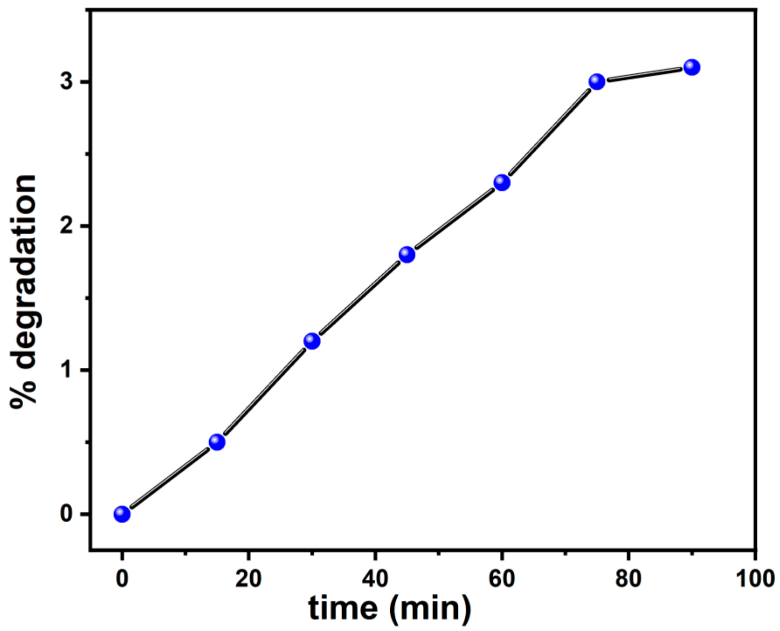

Figure S2. Plain photolysis of OFL under visible light.

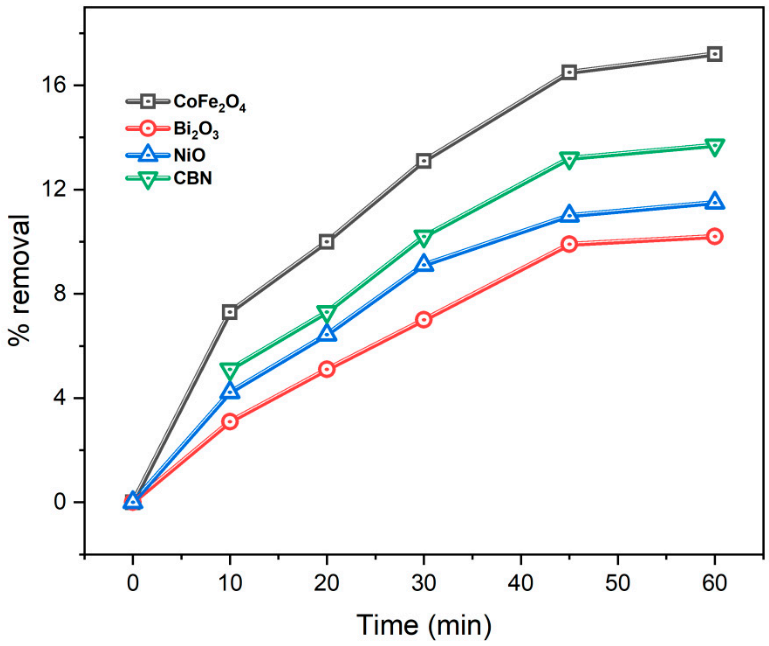

Figure S3. Adsorptional removal of OFL in dark.

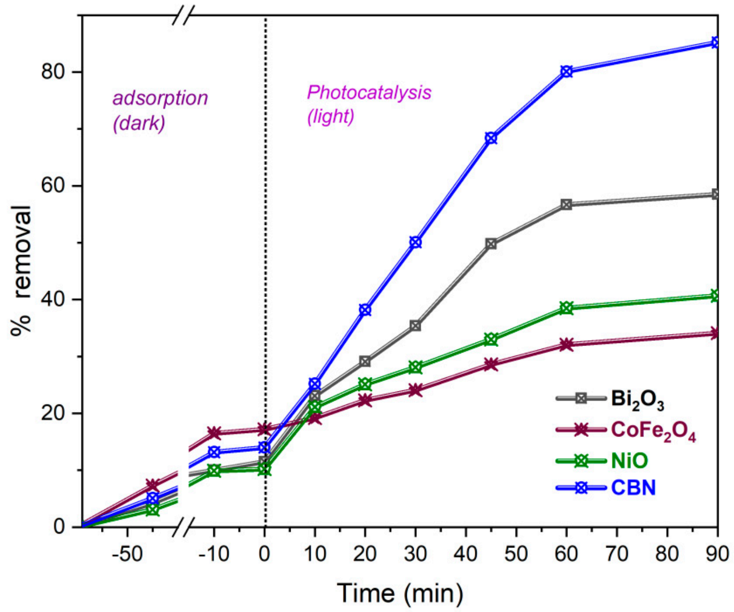

Figure S4. Removal of OFL: 60 min dark followed by 90 min visible light exposure.

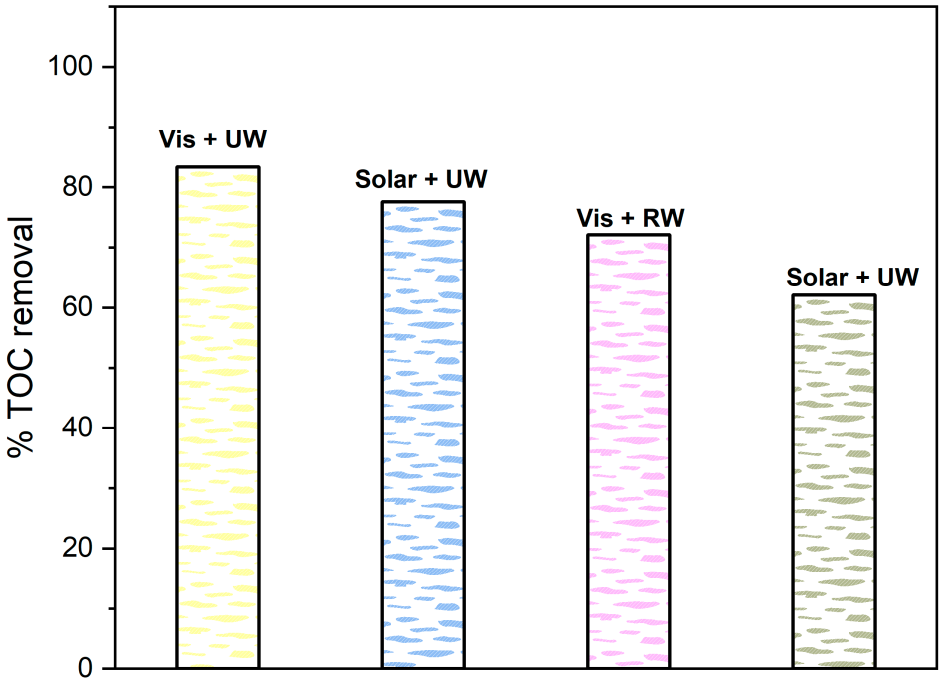

Figure S5. TOC removal during OFL degradation with CBN under visible light exposure.

Table S1. Elemental presence derived from XPS spectra for Bi_2O_3, NiO, andCBN.

XPS peak parameters for Bi_2O_3 sample				
**Name**	**Peak BE**	**FWHM eV**	**Area (P) CPS.eV**	**Atomic %**
**O1s**	530.4	2.98	1066199.51	45.77
**C1s**	285.15	2.91	343964.65	35.74
**Bi4f**	159.09	2.58	7363374.52	18.49

XPS peak parameters for NiO sample				
**Name**	**Peak BE**	**FWHM eV**	**Area (P) CPS.eV**	**Atomic %**
**O1s**	530.15	3.08	1221510.08	43.5
**Ni2p**	855.23	4.58	5247831.54	35.25
**C1s**	285.26	2.83	246434.75	21.25

XPS peak parameters for CBN sample				
**Name**	**Peak BE**	**FWHM eV**	**Area (P) CPS.eV**	**Atomic %**
**O1s**	530.05	2.9	1252119.31	43.95
**C1s**	285.17	3.06	329631.18	28.01
**Fe2p**	711.09	5.59	1433869.92	11.82
**Bi4f**	158.92	2.64	3968225.15	8.15
**Ni2p3**	855.2	3.96	444421.36	4.31
**Co2p**	780.56	3.13	541023.13	3.76
